# Telomerase Reverse Transcriptase (TERT) Expression, Telomerase Activity, and Expression of Matrix Metalloproteinases (MMP)-1/-2/-9 in Feline Oral Squamous Cell Carcinoma Cell Lines Associated With *Felis catus* Papillomavirus Type-2 Infection

**DOI:** 10.3389/fvets.2020.00148

**Published:** 2020-03-27

**Authors:** Gennaro Altamura, Manuela Martano, Luca Licenziato, Paola Maiolino, Giuseppe Borzacchiello

**Affiliations:** Department of Veterinary Medicine and Animal Productions, University of Naples Federico II, Naples, Italy

**Keywords:** telomerase, matrix metalloproteinases, cat, infection, oncogenes, oral squamous cell carcinoma

## Abstract

Telomerase activity contributes to cell immortalization by avoiding telomere shortening at each cell division; indeed, its catalytic subunit telomerase reverse transcriptase (TERT) is overexpressed in many tumors, including human oral squamous cell carcinoma (hOSCC). In these tumors, matrix metalloproteinases (MMPs), a group of zinc-dependent endopeptidases involved in cell migration, contribute to invasive potential of cancer cells. A proportion of hOSCC is associated with infection by high-risk human papillomavirus (HR-HPVs), whose E6 oncogene enhances TERT and MMPs expression, thus promoting cancer progression. Feline oral squamous cell carcinoma (FOSCC) is a malignant tumor with highly invasive phenotype; however, studies on telomerase activity, TERT, and MMPs expression are scarce. In this study, we demonstrate telomerase activity, expression of TERT, and its transcriptional activator cMyc along with expression of MMP-1, -2, and -9 in FOSCC-derived cell lines SCCF2 and SCCF3, suggesting a contribution by these pathways in cell immortalization and invasion in these tumors. Recent studies suggest that a sub-group of FOSCC as well as SCCF2 and SCCF3 are associated with *Felis catus* PV type-2 (FcaPV-2) infection. However, in this work, FcaPV-2 E6 gene knock-down caused no shift in either TERT, cMyc, or MMPs levels, suggesting that, unlike its human counterpart, the viral oncogene plays no role in their regulation.

## Introduction

Telomerase is a ribonucleoprotein enzyme complex whose main function is to extend telomeric DNA by adding repetitive sequences of six nucleotides (5′-TTAGGG-3′) at telomere ends (1). Telomerase reverse transcriptase (TERT) is the catalytic subunit and its activity consists in adding this six-nucleotide repeat using the RNA template (TR) included in the holoenzyme (1). TERT is not expressed in somatic cells (1). As a consequence, telomeric DNA is shortened at each cell division until reaching a critical point of erosion after a programmed number of cellular replications: this event is sensed by the cell machinery as severe DNA damage, leading to replicative senescence and induction of apoptosis. Therefore, telomerase activity is prominent in cells that must keep a high proliferative potential, such as stem cells and, importantly, neoplastic cells (1).

Indeed, TERT is expressed in most cancers, including human oral squamous cell carcinoma (hOSCC) (2). In these tumors, telomerase activity contributes to cellular immortalization, playing a key role in neoplastic process and representing a marker of poor prognosis (3, 4). A sub-group of hOSCCs, localized at oropharyngeal sites, are believed to be caused by high-risk human papillomavirus (HR-HPVs) infection, particularly HPV type 16 (HPV-16) (5). HPV-16 may contribute to activation of telomerase through viral oncoprotein E6, which is able to enhance TERT expression and increase enzymatic activity by different mechanisms such as promoter activation or epigenetic or post-transcriptional regulation (1).

cMyc is a well-known oncogene that contributes to tumorigenesis in different manners, such as regulation of genes involved in cell proliferation and apoptosis or mediating genomic instability (6, 7). It is overexpressed in many tumors, including hOSCC (8). Among the most relevant functions of cMyc, transcriptional activation of TERT gene is of great importance in neoplastic process, particularly in HPV-related cancer (9). Indeed, HPV-16 E6 is able to induce expression of cMyc, which, in turn, activates TERT promoter, thus contributing to cell immortalization (9).

Matrix metalloproteinases (MMPs) are a group of zinc-dependent endopeptidases that degrade basal membrane (BM) and extracellular matrix (ECM) (10). Most of the MMPs are synthetized as pro-active forms that need protease cleavage to become functional and exert their proteolytic activity toward connective tissues (10). This function is necessary for cells during several physiological processes, such as inflammation, embryogenesis, and wound healing; however, it is relevant also in pathological conditions, such as cancer (10). Degradation and remodeling of BM and ECM by cancer cells is a key step promoting invasiveness and metastasis; therefore, MMPs are expressed in many tumors, including hOSCC (10, 11). For instance, MMP-1, MMP-2, and MMP-9 (also known as collagenase, gelatinase A and B, respectively) are expressed and involved in cell migration and invasion as well as in malignant progression in hOSCC (12–14). In the case of HPV-related tumors, E6 and E7 oncogenes may play a role in inducing MMP-1, MMP-2, and MMP-9 expression, thus contributing to invasive phenotype of cancer cells (15–17).

In the field of pet oncology, TERT expression and telomerase activity have been described in many canine and feline tumors but poorly investigated in OSCC (18). In cats, OSCC is among the most common malignancies (19). It is characterized by highly malignant behavior, with frequent occurrence of local invasion and metastasis, high rate of recurrence, and poor prognosis (19). Feline OSCC (FOSCC) is considered a spontaneous animal model of human counterpart, since several biological properties are shared between tumors of the two species, including activation of cancer-related molecular pathways and prognostic markers (19, 20). However, there is only one report describing telomerase activity in FOSCC samples and studies regarding expression of TERT and its correlation with enzymatic activity in these types of cancer are lacking, particularly in tumor-derived living cells (21). Similarly, despite their high invasive potential, expression of MMPs in FOSCC has never been investigated so far.

It has been recently demonstrated that a sub-group of FOSCC is associated with *Felis catus* papillomavirus type-2 infection (FcaPV-2), whose oncogenes show transforming abilities comparable to those of HPV-16, thus mimicking HPV-related hOSCC (22–26). However, the possible role of FcaPV-2 E6 in the regulation of telomerase and MMPs is still unknown.

The aim of this study was to assess telomerase activity, expression of TERT, cMyc, MMP-1, MMP-2, and MMP-9 in FOSCC cell lines associated with FcaPV-2 infection. Moreover, the possible involvement of FcaPV-2 E6 in their regulation has been checked by viral gene knock-down approach.

## Methods

### Cells and Cell Culture

Cervical carcinoma Hela cells were purchased at ATCC cell bank. Feline oral squamous cell carcinoma cell lines SCCF2 and SCCF3 developed in the Rosol laboratory are a kind gift from Professor T. J. Rosol (The Ohio State University). SCCF2 have been obtained from gingival SCC with bone invasion, and SCCF3 have been obtained from a tongue lesion. Cells have been cultured as previously described (27–29).

### Telomeric Repeat Amplification Protocol (TRAP) Assay

Cells were plated in six-well-plates at 1 × 10^5^ density and harvested by trypsinization after 48 h. Telomerase activity in cells was assessed by a TRAP assay by using TRAPeze® Telomerase Detection Kit (Merck #S7700) following the manufacturer's protocol. Briefly, cell pellets were homogenized in CHAPS lysis buffer, incubated on ice for 30 min and centrifuged at 13,000 g for 20 min at 4°C. Supernatants were collected and the protein concentration was measured by Bradford assay (Bio-Rad Laboratories). The same amount of protein lysate (1.5 μg) was added to the reaction mixture (50 μl) and subjected to telomerase activation and amplification of telomerase products following the PCR protocol provided by the brand. Amplification products were separated by electrophoresis on 15% polyacrylamide non-denaturing gels. Gels were stained with GelStar™ Nucleic Acid Gel Stain (Lonza, #50535) and the ChemiDoc gel scanner (Bio-Rad) equipped with a densitometric workstation (Image Lab software, Bio-Rad) was used for quantification. Telomerase activity was calculated as the ratio between the telomerase ladders and the 36-base pair internal control.

### Western Blotting

Cell pellets were subjected to total protein extraction, protein quantification, sodium dodecyl sulfate (SDS)–polyacrylamide gel electrophoresis (PAGE) and Western blotting (WB) as described previously (30). Membranes were incubated with the following primary antibodies overnight (O/N) at 4°C at 1:1,000 dilution: anti-TERT (rabbit polyclonal, Rockland #600-401-252), anti-cMyc (mouse monoclonal, Santa Cruz Biotechnology, #sc-40, clone 9E10), anti-MMP-1 (mouse monoclonal, Santa Cruz Biotechnology, #sc-21731, clone 3B6) and anti-MMP-2 (rabbit polyclonal, Neomarkers, #RB-1537-P0), anti-MMP-9 (mouse monoclonal, Millipore, #MAB3309, clone 56-2a4), and β-actin (mouse monoclonal, Calbiochem #CP01-1EA, clone JLA20) or tubulin (mouse monoclonal, Santa Cruz Biotechnology, #sc-23948, clone B-5-1-2). After washing steps in Tris-buffered saline (TBS) 0.1% Tween-20 (TBST), goat anti-mouse (GE Healthcare #LNA931V/AH), and donkey anti-rabbit (Bethyl Laboratories #A-120-108P) secondary antibodies conjugated with horseradish peroxidase (HRP) were applied for 1 h at room temperature (rt). Following further washings in TBST, protein bands were visualized by enhanced chemiluminescence (ECL, Clarity ECL Western Blotting Substrate, Bio-Rad). Quantization of bands was performed by densitometric analysis using ChemiDoc gel scanner (Bio-Rad) equipped with a densitometric workstation (Image Lab software, Bio-Rad). Protein expression levels were normalized to β-actin or tubulin.

### RNA Extraction, Reverse Transcription (RT), and Real-Time Quantitative PCR (qPCR)

Cells were subjected to RNA extraction by using the RNeasy Mini Kit (Qiagen #73504) followed by DNase digestion (Roche, #04536282001) according to manufacturers' recommendations. For each sample, 1 μg of RNA was subjected to RT by using iScript cDNA Synthesis Kit (Bio-Rad Laboratories, #1708890); RT without the addition of reverse transcriptase enzyme was also performed on each RNA sample as control. Real-time qPCR was performed on 50 ng of the obtained cDNAs by using iTaq Universal SYBR Green Supermix (Bio-Rad Laboratories, #1725121) according to the brand instructions, employing the primers for feline TERT and FcaPV-2 E6 detailed elsewhere (31, 32). The following primers for feline cMyc were designed based on the gene sequence previously published: cMyc-FW: 5′-CAAAAGGTCGGAATCGGGGT-3′, cMyc-REV: 5′-CGTGGCATCTCTTAAGGACCA-3′ (33). Feline MMP-1, MMP-2, and MMP-9 genes were amplified by using the primers described previously (34–36). Amplification of feline β-2-microglobulin (β2MG) was performed in parallel to allow normalization of the results as previously reported (37). Bio-Rad CFX Manager software was used to generate gene expression data based on the 2^−ΔΔ*Ct*^ method, and SCCF2 were arbitrarily set as control and put as 1.

### Immunofluorescence Staining

Cells grown for 48 h on coverslips were washed, fixed, permeabilized, and subjected to background blocking as previously reported (30). Primary antibodies anti-TERT, anti-MMP-2, and anti-MMP-9 at 1:50 dilution in PBS were applied for 2 h at rt in a humid chamber. Anti-MMP-1 antibody does not work for immunofluorescence. Then, slides were washed three times for 10 min in PBS and incubated with Texas Red Alexa Fluor goat anti-rabbit (Thermo Fisher Scientific #A11030) and Alexa Fluor 488 goat anti- mouse (Thermo Fisher Scientific #A11001) for 30 min at rt in a humid chamber at 1:100 dilution. Finally, after washing with PBS, the slides were mounted in aqueous medium PBS:glycerol 1:1 containing DAPI (1:1,000) to allow nuclear counter-staining. Slides were read under ZOE Fluorescent Cell Imager (Bio-Rad Laboratories) for scanning and photography.

### FcaPV-2 E6 Gene Knock-Down by siRNA

FcaPV-2 E6 gene silencing was achieved by using three custom-synthetized siRNA oligonucleotides (Silencer@ Select, Ambion #4399666) as previously described (24). SCCF3 cells were plated at 2 × 10^5^ density in six-well-plates and, after 24 h, a siRNA oligonucleotides pool or scrambled RNA (Ambion #4390843) at 50 nM were transfected by using Lipofectamine 2000 (Thermo Fisher Scientific #11668027) according to the standard protocol. Cells were harvested after 48 h and analyzed by WB and qPCR.

### Statistical Analysis

For statistical analysis, Student's *t*-test was performed using SPSS 17.0 software (SPSS Inc., Chicago, ILL, USA) and differences considered statistically significant for ^*^P < 0.05 or ^**^P < 0.01.

## Results

### Expression of TERT and cMyc in FOSCC Cell Lines

**Figure 1:**
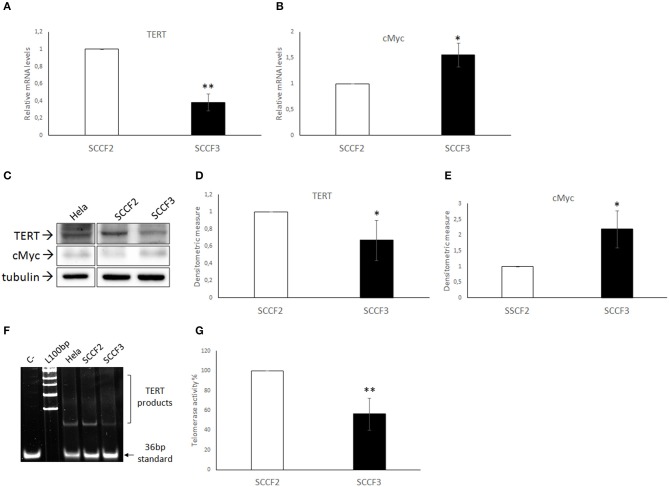
Expression of TERT, cMyc, and telomerase activity in SCCF2 and SCCF3 **(A,B)** TERT and cMyc gene expression in SCCF2 and SCCF3. Data are normalized for β2-microglobulin expression as housekeeping gene and expressed as relative quantization by the 2^−ΔΔCt^ method. Relative mRNA levels of TERT were lower while cMyc gene expression was higher in SCCF3 with respect to SCCF2 in at least three repeated, independent experiments. **(C)** A representative Western blotting (WB) gel showing lower TERT protein levels but higher cMyc amounts in SCCF3 vs. SCCF2 is illustrated. Hela whole cell lysate run along with feline samples confirmed the identity of the band. The blot was stripped and reprobed for tubulin to ensure comparable protein loading and allow normalization. Boxes are cut from the same gel at the same exposure time and properly aligned according to molecular standards loaded onto the gel. Full scans from original gels are shown in Supplementary Figure 2. **(D,E)** Mean densitometric values ± SD from at least three repeated, independent WB experiments, normalized for tubulin expression (statistically significant, *P < 0.05 and **P < 0.01). **(F)** Telomerase activity was detected in SCCF2 and SCCF3 by telomeric repeat amplification protocol (TRAP) assay. Hela cell lysate was run along with feline samples as positive control. A representative gel out of four independent experiments, showing lower telomerase activity in SCCF3 vs. SCCF2 is illustrated (C–: negative control, sample with no lysate; L100bp: 100 base pairs DNA ladder, the first band from the bottom is 100 bp). **(G)** Quantification of telomerase activity in SCCF2 and SCCF3 by densitometric analysis expressed in % relative to SCCF2. Data were calculated as the ratio between TERT products ladder and 36 bp internal standard and represent the mean ± standard deviations (SD) of four repeated, independent experiments (statistically significant, **P < 0.01).

Expression of telomerase catalytic subunit TERT is crucial for enzymatic activity (1). Therefore, expression of TERT and its transcriptional activator cMyc were investigated at gene and protein levels in SCCF2 and SCCF3 by real-time qPCR and WB, respectively (Figure 1). TERT and cMyc cDNA were successfully amplified in both cell lines by qPCR; relative quantization analysis revealed lower TERT gene expression but higher cMyc relative mRNA levels in SCCF3 compared to SCCF2 (Figures 1A,B). The data obtained by WB followed by densitometric analysis yielded consistent results, showing lower TERT protein expression but higher cMyc protein amounts in SCCF3 with respect to SCCF2 (Figures 1C–E). Hela whole cell lysate run along with feline samples as antibody control confirmed the identity of the band (38). All the experiments were repeated at least three times yielding comparable results and the differences detected between the SCCF2 and SCCF3 cell lines were statistically significant (*t*-test).

### Telomerase Activity in FOSCC Cell Lines

Then, telomerase activity was investigated by TRAP assay in SCCF2 and SCCF3 (Figure 1F). Results from four repeated, independent experiments showed that telomerase was active in both cell lines, with lower enzymatic activity in SCCF3 with respect to SCCF2 (Figure 1G). As expected, telomerase activity was detected also in Hela used as positive control but not in the sample with no lysate run as negative control [Figure 1F; (38)].

### Expression of MMP-1, MMP-2, and MMP-9 in FOSCC Cell Lines

**Figure 2:**
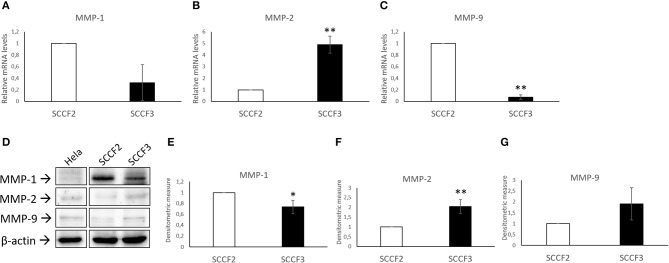
Expression of MMP-1, MMP-2, and MMP-9 in SCCF2 and SCCF3 cell lines. **(A–C)** MMP-1, MMP-2, and MMP-9 gene expression in SCCF2 and SCCF3. Data are normalized for β2-microglobulin expression as housekeeping gene and expressed as relative quantization by the 2^−ΔΔCt^ method. Relative mRNA levels of MMP-1 and MMP-9 were lower while MMP-2 gene expression was higher in SCCF3 with respect to SCCF2 in at least three repeated, independent experiments. **(D)** Representative Western blotting (WB) panels showing variable expression levels of MMP-1, MMP-2, and MMP-9 in cell lysates. Hela whole cell lysate run along with feline samples confirmed the identity of the bands. The blot was stripped and reprobed for β-actin to ensure comparable protein loading and allow normalization. Boxes are cut from the same gel at the same exposure time and properly aligned according to molecular standards loaded onto the gel. Full scans from original gels are shown in Supplementary Figure 3. **(E–G)** Quantitative analysis expressed as mean densitometric values ± standard deviations from at least three independent experiments revealed lower MMP-1 levels and higher MMP-2 and MMP-9 protein amounts in SCCF3 compared to SCCF2. Protein bands were normalized for β-actin expression (statistically significant, *P < 0.05 and **P < 0.01).

Expression of MMP-1, MMP-2, and MMP-9 was also checked by qPCR (Figure 2). Relative quantization analysis showed lower MMP-1 and MMP-9 but higher MMP-2 mRNA levels in SCCF3 with respect to SCCF2 (Figures 2A–C). By WB, protein bands of the investigated MMPs were detected in both cell lines (Figure 2D) and, consistently with gene expression data, densitometric analysis confirmed lower MMP-1 and higher MMP-2 expression in SCCF3 compared to SCCF2 (Figures 2E,F). Surprisingly, MMP-9 was detected at higher protein levels in SCCF3 with respect to SCCF2 in contrast with gene expression results (Figure 2G). The identity of the bands was confirmed in Hela cell lysate run as antibodies control (39). The experiments were repeated in triplicate and the differences yielded by densitometric analysis were statistically significant as revealed by *t*-test.

### Sub-cellular Localization of TERT and MMPs in FOSCC Cell Lines

**Figure 3:**
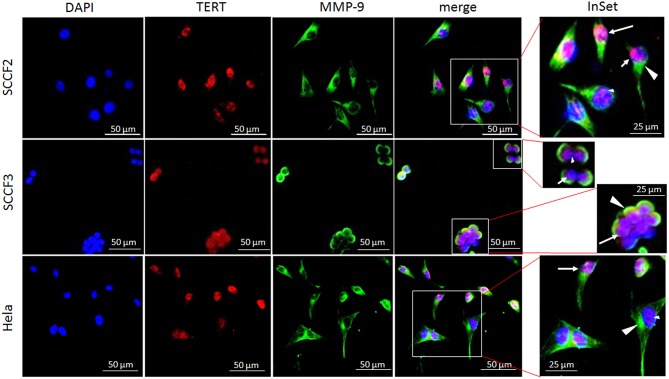
Sub-cellular localization of TERT and MMP-9 in SCCF2 and SCCF3. Cells were grown on coverslips and subjected to double indirect IF staining for TERT (red fluorescence) and MMP-9 (green fluorescence). Nuclei were counterstained with DAPI. Inset shows higher magnification of merge panel. TERT was localized in the whole nuclear area (long arrow), compartmentalized in proximity of nuclear membrane (short arrow) or in dot-like spots (small arrowhead). MMP-9 was expressed in the cytoplasm (large arrowhead). Hela were stained to ensure antibody reactivity.

Sub-cellular localization of TERT is functionally relevant for telomerase activity; therefore, SCCF2 and SCCF3 were subjected to double IF staining for TERT, MMP-2, and MMP-9, along with Hela cells to ensure the reactivity of the antibodies [Figure 3; (40)]. TERT (red staining) was localized mainly in the nuclei in all the cell lines, as judged by the merge with DAPI blue labeling. In SCCF2, SCCF3, and Hela cells, TERT staining showed three different localization patterns: diffused to the whole nuclear area, compartmentalized in proximity of the nuclear membrane or with dot-like spots (Figure 3).

MMP-9 (green staining) was localized in the cytoplasm of SCCF2, SCCF3, and Hela cells (Figure 3). Cytoplasmic staining was also observed for MMP-2 (not shown).

### Effects of FcaPV-2 E6 Knock-Down on TERT, cMyc, and MMP Expression

**Figure 4:**
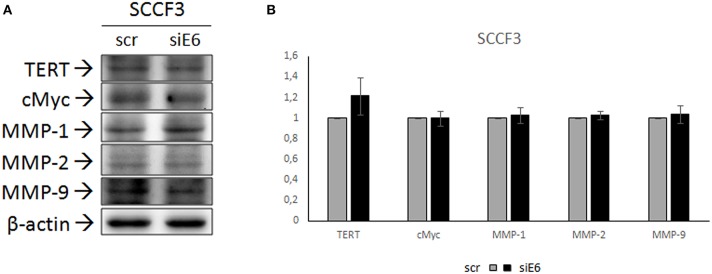
Effects of FcaPV-2 E6 gene silencing on TERT, cMyc, and MMP expression in SCCF3 cell line. **(A)** Representative WB panels showing no changes in TERT, cMyc, MMP-1, MMP-2, and MMP-9 protein levels upon FcaPV-2 E6 gene knock-down siRNA (siE6) vs. scramble RNA (scr) treatment. The blot was stripped and reprobed for β-actin to ensure comparable protein loading and allow normalization. **(B)** Mean densitometric values normalized for β-actin expression ± standard deviations out of two independent experiments.

FOSCC cell lines employed in this study had been shown to express FcaPV-2 E6 oncogene, particularly at higher levels in SCCF3 (24). In order to investigate whether expression of TERT, cMyc, and MMPs might be dependent on expression of this viral oncogene, SCCF3 were subjected to E6 knock-down by siRNA, followed by WB and densitometric analysis to evaluate changes in their protein levels. Moreover, given that FcaPV-2 E6 is known to degrade p53, cells were concomitantly analyzed by WB with anti-p53 antibody to evaluate p53 rescue and ensure the reliability of the procedure (23, 24). As expected, qPCR confirmed knock-down of E6 expression along with accumulation of p53, as revealed by WB (Supplementary Figure 1). However, no shift in protein expression of TERT, cMyc, MMP-1, MMP-2, and MMP-9 compared to scramble-treated cells could be appreciated (Figure 4A). Densitometric analysis confirmed these results (Figure 4B).

## Discussion

Telomerase is an enzyme that contributes to immortalization in cancer cells (1). Its catalytic subunit TERT is overexpressed in many human and animal tumors; however, studies regarding expression of TERT and telomerase activity in FOSCC are scarce (18, 31). Here, we show, for the first time, TERT expression and telomerase activity in FOSCC-derived cell lines SCCF2 and SCCF3, in agreement with a unique study reporting functional enzymatic activation in FOSCC samples: this suggests that telomerase may play a role in neoplastic process in these tumors as shown in human and canine counterpart (2, 3, 21, 41). In cancer, different factors may influence the degree of telomerase activity, such as expression levels of TERT, TERT sub-cellular localization, or tissue origin of the lesion (40, 42). TRAP assay showed that the levels of telomerase activity were correlated with respective TERT mRNA and protein amounts in each cell line: this may indicate that the activation status of the enzyme is mostly dependent on expression levels of its catalytic subunit in FOSCC, differently from other types of cancer where it may be influenced also by post-translational events (43). It has been shown that telomerase exerts lower enzymatic activity when TERT is localized in proximity of nuclear membrane or compartmentalized in nuclear spots, while a nuclear diffuse expression pattern is associated with higher functional activation (40). In this study, IF staining showed all the aforementioned staining patterns concomitantly in both cell lines, suggesting that telomerase activity is not correlated with TERT sub-cellular localization in FOSCC. These diverse intra-nuclear locations are possibly related with different steps of complete telomerase assembly; however, it is not fully understood how TERT localizations may affect levels of telomerase activity (40). In hOSCC, tongue location is associated with higher telomerase activity with respect to gingival lesions (42). This might be not the case of FOSCC, since lower telomerase activity was detected in tongue SCCF3 vs. gingival SCCF2 cells, suggesting a biological discrepancy with human counterpart. Further studies are needed to clarify this point.

cMyc is a potent oncogene that is overexpressed in many tumors (6, 7). Its key role in cancer progression is confirmed by the fact that expression levels of cMyc are closely correlated with chemotherapy resistance in different types of malignancies (44). This might be plausible also in FOSCC, since SCCF3 harboring higher cMyc amounts had displayed lower sensitivity to several chemotherapeutics with respect to SCCF2, as described in a recent work (45). A possible role of cMyc in FOSCC development was further suggested in an elder report showing its overexpression in tumor samples, consistently with our results (46).

In humans, a sub-group of OSCC is associated with HR-HPV infection (5). In these tumors, HPV-16 E6 oncoprotein switches on immortalization pathways, among these telomerase activity (1). The most relevant mechanism of telomerase activation by HPV-16 E6 is the augmentation of TERT gene expression through multilayered functions (1). For instance, HPV-16 E6 induces overexpression of cMyc, which is a transcriptional activator of TERT promoter (9). Recent studies demonstrate that a proportion of FOSCCs is associated with FcaPV-2 infection; importantly, FcaPV-2 biological activity has been previously revealed also in SCCF2 and SCCF3, with this latter cell line harboring higher E6 mRNA amounts (24, 26). In this study, expression levels of cMyc, but not TERT, appeared to be correlated with those of E6 in SCCF cells; at first glance, these data suggested that the viral oncogene may up-regulate cMyc as in human counterpart, but this would not contribute to cell transformation through induction of TERT expression in FcaPV-2-related FOSCC. However, E6 siRNA did not affect neither cMyc nor TERT protein amounts, indicating that their expression was independent from the viral oncogene. It has been shown that TERT expression may be induced by promoter mutations independently from the HPV status in hOSCC and cervical cancer; whether this may occur in FOSCC has to be further investigated in future studies, in order to shed light on the mechanisms leading to TERT expression and telomerase activation in these tumors (47).

MMPs are proteolytic enzymes produced by cancer cells to digest ECM and BM in order to promote local invasion and metastasis (10). FOSCCs are characterized by high invasive behavior, particularly at bone level (19). SCCF2 and SCCF3 showed expression of MMP-1/-2/-9 at gene and protein level, suggesting that they might contribute to invasive potential of FOSCC cells, in agreement with studies on hOSCC that demonstrate that these MMPs play a relevant role in determining the invasive phenotype of tumor cells (11). In the case of MMP-9, the inconsistency between gene and protein expression data could be due to well-known post-transcriptional and/or post-translational regulation mechanisms that can cause discrepancy between the steady-state levels of its mRNA and protein (48). A previous work reports that SCCF2 cells display major osteolysis and bone invasion with respect to SCCF3 (29). Therefore, higher gene and protein expression of MMP-1 in SCCF2 found in the current study might indicate that it may be the main factor responsible in influencing bone invasiveness in FOSCC. MMP-2 and MMP-9 are classified as gelatinases A and B, respectively (11). MMPs belonging to this class are produced as zymogens that are stored in the cytoplasm to be released and activated in the extracellular environment (11, 49). Thus, MMP-2 and MMP-9 cytoplasmic staining revealed by IF in this study is likely to represent their intracellular storage in SCCF2 and SCCF3 and is consistent with the scenario described in cell lines derived from hOSCC (50). In veterinary oncology, overexpression of MMP-2 and MMP-9 has been reported in equine sarcoid associated with bovine PV infection (51). Moreover, HR-HPV types associated with hOSCC may enhance expression of MMP-2 and MMP-9 through the molecular activity of E6 oncogene, thus promoting invasiveness (16). In our study, higher amounts of MMP-2 in SCCF3 suggested a possible functional correlation with FcaPV-2 E6 expression; however, siRNA for viral gene transcript did not affect MMPs protein levels, suggesting a biological difference with other PV-related tumors. Other viral-independent pathways have been shown to induce MMP-1, MMP-2, and MMP-9 expression in hOSCC, among these intracellular signaling activated by pro-inflammatory cytokines (11, 50). Recent studies are highlighting a possible role of inflammation also in FOSCC progression; therefore, this field of inquiry should be deepened in future studies (52, 53).

Previous studies revealed that SCCF2 and SCCF3 are highly representative of spontaneous tumors regarding invasiveness and cancer-related pathways (29, 54). Therefore, expression of TERT and cMyc, telomerase activity, and MMP-1/-2/-9 demonstrated in this study confirm the reliability of these cell lines as a valuable preclinical model of FOSCC. FcaPV-2 E6 seems to play no role in their regulation, differently from its human counterpart; however, a possible involvement of other putative viral oncogenes cannot be excluded. Further studies are needed to clarify the role of TERT and MMPs in FOSCCs, as well as the possible involvement of FcaPV-2 in immortalization and invasion pathways in these tumors.

## Data Availability Statement

The datasets generated for this study are available on request to the corresponding author.

## Author Contributions

GA, MM, and LL performed the experiments. GA, MM, PM, and GB conceived the study and drafted the manuscript.

### Conflict of Interest

The authors declare that the research was conducted in the absence of any commercial or financial relationships that could be construed as a potential conflict of interest.
