# Supplementary material for: Telomerase Reverse Transcriptase (TERT) Expression, Telomerase Activity, and Expression of Matrix Metalloproteinases (MMP)-1/-2/-9 in Feline Oral Squamous Cell Carcinoma Cell Lines Associated With Felis catus Papillomavirus Type-2 Infection
Source: Front Vet Sci. 2020 Mar 27;7:148. doi: 10.3389/fvets.2020.00148 (PMC7118734; doi:10.3389/fvets.2020.00148)
Supplement: Supplementary file 1 [file Data_Sheet_1.PDF]

Supplementary Material

1 Supplementary Figure 1

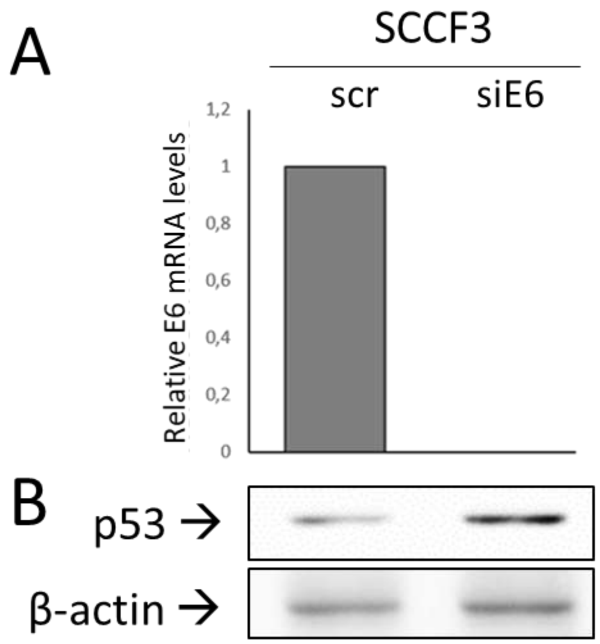

Supplementary Figure 1. FcaPV-2 E6 gene knock-down and rescue of p53. **(A)** qPCR data showing of FcaPV-2 E6 gene knock-down upon siRNA (siE6) *vs* scramble RNA (scr) treatement. **(B)** Western blotting showing rescue of p53. The blot was stripped and reprobed for β-actin to ensure equal protein loading.

2 Supplementary Figure 2

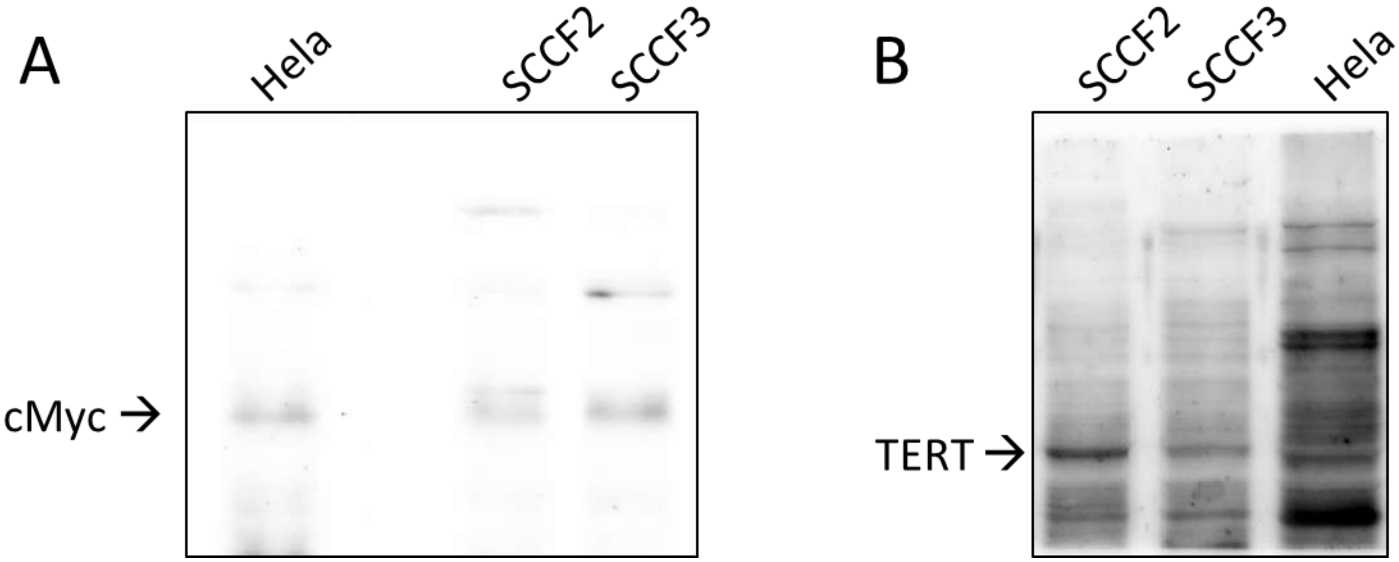

Supplementary Figure 2. Full scans from original gels of Western blotting experiments for cMyc **(A)** and TERT **(B)** shown in Figure 1.

3 **Supplementary Figure 3**

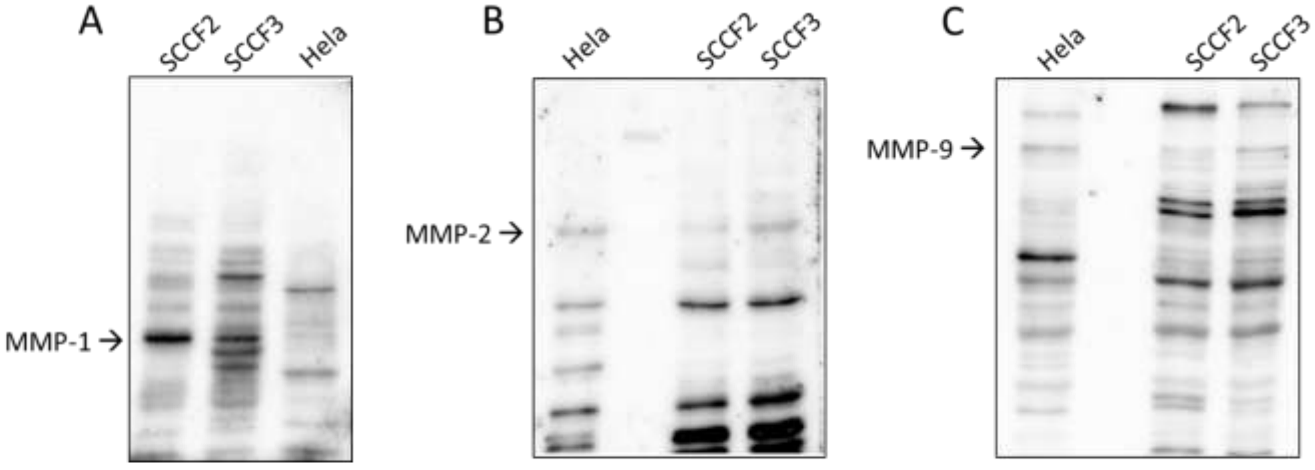

Supplementary Figure 3. Full scans from original gels of Western blotting experiments for MMP-1 **(A)**, MMP-2 **(B)** and MMP-9 **(C)** shown in Figure 2.
